# A Multimodal Sequence-to-Sequence Model for Automatic Assignment of ATC Codes in Drug Discovery and Repurposing

**DOI:** 10.1021/acs.jcim.6c00118

**Published:** 2026-04-14

**Authors:** Trinidad Crozes, Eugenia Ulzurrun, Juan A. Páez, Nuria E. Campillo, Axel J. Soto, Ignacio Ponzoni

**Affiliations:** † Institute for Computer Science and Engineering, 541419 UNS-CONICET, 8000 Bahía Blanca, Argentina; ‡ Department of Computer Science and Engineering, 54446 Universidad Nacional del Sur, 8000 Bahía Blanca, Argentina; § Centro de Investigaciones Biológicas Margarita Salas (CIB Margarita Salas-CSIC), C/Ramiro de Maeztu, 9, 28040 Madrid, Spain; ∥ Instituto de Química Médica (IQM-CSIC), C/Juan de la Cierva, 3, 28006 Madrid, Spain

## Abstract

The Anatomical Therapeutic Chemical (ATC) code is a drug classification system that indicates the therapeutic potential use of a compound. Predicting ATC codes for drugs using automatic approaches is key to guide clinical trials and for drug repurposing. However, such automatic assignment is challenging due to the hierarchical organization of the code in four levels, possible polypharmacological behavior, and the imbalance and scarcity of annotated data in relation to the large number of compounds and possible ATC codes a drug may have. In this work, we propose a novel multimodal generative approach for predicting ATC codes, which leverages molecular information using a sequence-to-sequence architecture. Our hypothesis explores the idea that describing the chemical structure of the input compounds using two different representations, i.e., modes, the SMILES code and its molecular descriptors, provides complementary information, hence improving the accuracy of the predictions. Furthermore, given the multilabel nature of generative sequence-based models, we also present an additional prediction method to determine when to stop generating ATC labels for each compound. We compared the performance of our proposed methods against several baselines, both for new drugs and in drug repurposing tasks. In all of these cases, the superior performance of our multimodal proposals is clearly demonstrated. The source code and different data sets used to train and evaluate the models are made publicly available.

## Graphical abstract

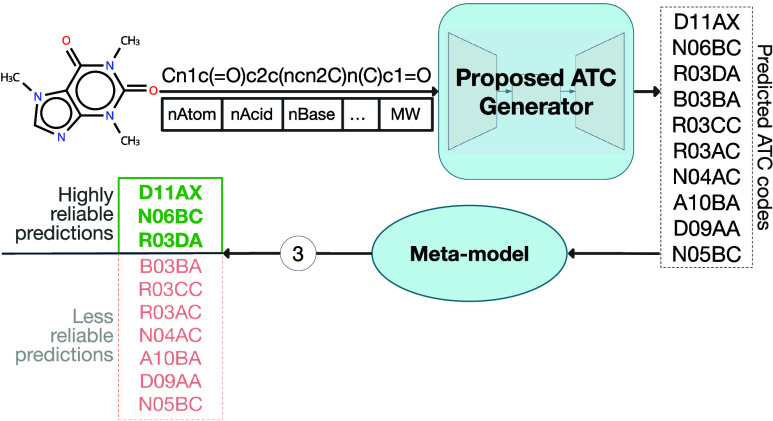

## Introduction

Drug discovery is a laborious, complex, and costly process. The methods for the discovery of new hits have ranged from the most basic ones, such as blind screening, to the application of mathematical models, computational chemistry, and, more recently, the use of artificial intelligence (AI). All of these strategies have historically contributed to the development of new drugs with therapeutic applications.

Drug development can be likened to an obstacle race in which many competing molecules start, but only a few cross the finish line.^1^ On average, it takes between 10 and 12 years for a drug to reach the market, with development costs reaching up to US$2–3 billion.^2,3^ In addition, the complexity of this process is accentuated by a high failure rate, with approximately 90–95% of drug candidates evaluated throughout this process never reaching the market. Therefore, any strategy that can shorten the time required for drug development and, in this way, reduce the investment needed to bring a drug to the market would be very important.

In these circumstances, an early understanding of the therapeutic potential use of a compound becomes critical not only for optimizing development strategies but also for facilitating downstream applications, such as classification, regulatory assessment, and clinical integration. In this context, the ATC (Anatomical Therapeutic Chemical) code,^4^ which categorizes drugs according to their target organ or system, therapeutic intent, and chemical properties, plays a central role in this process. Accurately assigning ATC codes early on can support prioritization decisions, guide clinical trial design, and ultimately help identify potential avenues for drug repurposing, thus increasing the chances of success in the highly competitive and risky landscape of pharmaceutical innovation. In particular, drug repurposing, also known as drug repositioning, involves the investigation of existing drugs for new therapeutic purposes and allows the development of a drug for approximately $300 million in a time frame of 6.5 years and a 40% success rate.^3^

The ATC classification system (Figure 1), developed by the World Health Organization (WHO), provides a hierarchical framework for classifying drugs according to the organ or system on which they act and their therapeutic, pharmacological, and chemical properties.^5^ The first level identifies the primary anatomical group, indicating the organ or system primarily targeted by the drug (e.g., the alimentary tract and metabolism). The second level defines the therapeutic sublevel, which describes the drug’s primary use or therapeutic purpose (e.g., drugs for acid-related disorders). The third level classifies the pharmacological sublevel according to its mechanism of action (e.g., drugs for peptic ulcer and gastroesophageal reflux disease). Finally, the fourth level specifies the chemical sublevel, grouping drugs with similar chemical structures or closely related properties (e.g., proton pump inhibitors). Therefore, each drug is assigned one or more ATC codes that reflect its therapeutic use, enabling standardized communication across clinical, pharmacological, and regulatory contexts.

**Figure 1:**
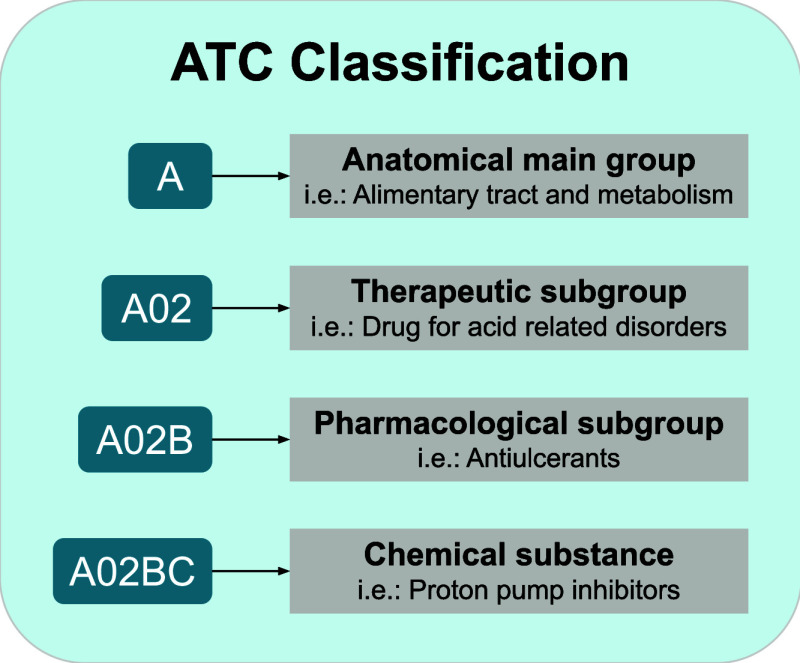
ATC classification system developed by the WHO. The figure shows an example of a code with meaning at each of the first four levels.

From a practical point of view, the ATC classification system provides a globally standardized taxonomy that facilitates communication, data comparison, and interoperability in pharmacology and medicine by offering a universal reference framework that is used by researchers, clinicians, and regulatory agencies worldwide. It supports biomedical research by enabling prescription pattern analysis, drug utilization studies, outcome-based evaluations, and the identification of repurposing opportunities based on therapeutic and pharmacological similarities. In pharmacovigilance, ATC codes allow systematic monitoring of drug use, early detection of safety signals, and harmonization of surveillance databases across countries and institutions. For clinical management and health policy, ATC coding ensures unambiguous drug identification, enables international comparisons, supports evidence-based policymaking, and enhances data integration across health information systems. In education and training, the hierarchical ATC structure helps learners and professionals understand pharmacological relationships and clinical applications in a coherent and accessible way.

Despite their utility, the manual assignment of ATC codes remains a complex and labor-intensive task, typically requiring expert domain knowledge and an extensive review of pharmacological data. This process becomes increasingly difficult, with the growing number of new drugs and compound variations entering the market. Moreover, many compounds exhibit polypharmacological behavior or off-label effects that challenge rigid classification based solely on primary indications.

For the reasons stated above, several proposals naturally arose to employ computational models for assisting experts in the assignment of ATC codes for new compounds and for repurposing applications.^6^ However, the design of a computational approach to this problem presents significant challenges derived from the hierarchical nature of the classification system, which expands to a greater number of classes as we move forward along the ATC code levels, while the number of drugs annotated in the classes associated with each level decreases accordingly. This results in a limited number of samples available for the supervised learning of a complex multiclass classification system. Furthermore, since a single compound can have more than one associated ATC code, the problem becomes a multilabel classification task, which increases the difficulty of training an AI model.

In this paper, we present the development of novel multiclass and multilabel generative approaches for predicting ATC codes, based exclusively on the chemical structure of compounds, where the impact of using multimodal approaches is addressed. In this sense, our hypothesis is that the incorporation of the chemical structure from two different representations (modes), i.e., the SMILES code and molecular descriptors of the input compound, can contribute to better capturing the context of the information, thanks to the complementarity of these representations, thus potentially helping improve the accuracy of predictions. Our approach applies a sequence-to-sequence generative strategy that aims to learn how to “translate” the chemical structure into an ATC code using bidirectional LSTMs^7^ and Transformers.^8^ Given the generative characteristic of the proposed models, an additional prediction strategy, i.e., a meta-model, is also presented for deciding how many ATC labels should be generated as output for each compound. Regarding the hierarchical level of ATC labels to be predicted, we report the results achieved level by level since different practitioners may prefer to use these models with different purposes, and depending on the semantic implications of their application cases, they could need to predict ATC codes up to different levels. Finally, the source code and data sets used to train all models and perform the experiments presented in this paper are made available.

## Related Work

Previous studies have explored the prediction of ATC codes and identified several challenges associated with this task. This has led researchers to consider different strategies to tackle data scarcity conditions, ranging from restricting the ATC code level they aim to predict, transforming the problem into a binary or even single-label classification task, to incorporating other data sources (in addition to the chemical structure of the compound) to improve performance.

For a better discussion of prior research, we organized existing approaches taxonomically by proposing four criteria associated with their main design characteristics. This classification provides insight into how previous studies dealt with the complexity of predicting ATC codes and provides a framework for discussing our proposed contributions.

One common strategy to simplify the problem, followed in many studies, is to focus solely on predicting the first level of ATC codes. Therefore, a first criterion for taxonomic classification is associated with the hierarchical levels (or tiers) that each method aims to predict. As mentioned above, ATC codes classify drugs into hierarchical levels based on their anatomical, therapeutic, pharmacological, and chemical characteristics. Most computational approaches predict only the first ATC level, while others extend their predictions up to the second or third level. Several authors stated that predicting only the first level of the ATC code has limited applicability in real-world scenarios, while predicting up to the second or third levels of the ATC code is more valuable.^9^ In this sense, Zhang et al.^10^ proposed a molecular graph-based approach designed to enhance representation learning and tackle the low-data regime in the ATC code prediction task to the second level with reasonable performance metrics. However, in many contexts, prediction up to at least the third level is necessary.^9^ For example, Gurulingappa et al.^9^ present a concept-based classification system, whose performance was evaluated for a subset of drugs with ATC codes and indications for cardiovascular diseases—this involves predicting ATC codes beginning with the letter C. Semantically, in this case, ATC codes at the second level are very general, such as antihypertensives (C02) or diuretics (C03). However, class labels of the third and fourth levels define more specific and valuable pharmacological or chemical properties of drugs. However, since the class labels of the fourth level have an insufficient number of drug instances per class for training models, the authors decided to predict the class labels up to the third level since they showed optimal information coverage as well as sufficient samples per class to support a supervised learning process. More recent methods have attempted to predict the full ATC code up to the fourth level, although not all of them report the prediction accuracy separately for each ATC level.

The second criterion refers to the type of classifier used to assign the ATC code. While this problem is clearly a *multiclass* classification task, several works have transformed the problem of predicting ATC codes into a binary prediction task from drug-code pairs. In these methods, a drug is paired with a user-selected ATC code and fed into the supervised model to predict whether the ATC code can be assigned to the drug or not, hence transforming a multiclass and multilabel problem into a binary classification task. In practical terms, to build such a model, the training data set has to be extended with “negative” pairs, comprising a drug paired with ATC codes that have not been assigned to it. However, models trained on such imbalanced data sets may tend to predict the “negative” class rather than accurately identify the ATC properties associated with a specific drug. Consequently, this kind of methods are likely to fail at unknown drug repurposing scenarios.^10^

A third criterion relates to the number of output labels returned by the method. Several early methods predicted only a single label for a compound,^9,11,12^ which limits the possibility of finding new purposes for known drugs. Afterward, most researchers have sought to extend the prediction problem to multiple labels. For example, some works have adapted the multilabel problem using multiple independent binary classifiers,^13,14^ e.g., by determining whether a drug belongs to “antifungal” or “antiviral”, without considering the potential interactions between these labels. Other ensemble-based methods use label powerset approaches.^15,16^ Nevertheless, the current trend is oriented toward applying multilabel deep learning frameworks.^10,17–20^

The fourth criterion refers to the input data used to predict the ATC code of a compound: only structural information and/or basic physicochemical properties derived directly from the chemical structure of the compounds, or multiple data sources, incorporating additional information such as interactions with other compounds, their therapeutic targets, or side effects, among other types of data. Regarding this criterion, while the inclusion of new data sources can enrich molecular representations and help improve prediction, it can also introduce additional new challenges. One minor challenge is that, as the complexity of the representations and models increases, greater processing power is usually required to train and infer using these methods. More importantly, most of these new data are interactions extracted from STITCH,^21^ a data set compiled from previous clinical trials, physicochemical experiments, and meta-analyses. The reliance on STITCH makes the prediction of ATC dependent on prior laboratory experiments and therefore less feasible and practical for new or unpublished compounds.^19^ Researchers have recognized similar limitations, i.e., dependency on knowing the target proteins and side effects of a drug to generate reliable predictions of its ATC codes.^22,23^ Furthermore, recent contributions by Cao et al.^19^ and Zhang et al.^10^ showed that state-of-the-art performance levels can be achieved using only chemical structure information from compounds as input for the classifiers, eliminating the dependence on additional experiment-based resources. A summary of the published ATC prediction methods following this taxonomical classification is shown in Table 1.

**Table 1:** Taxonomical Classification of ATC Prediction Methods

method	pub. date	ATC levels	classifier	labels	input data
SuperPred^11^	May, 2008	up to fourth level	multiclass	single	chemical structure
Concept Based^9^	Aug, 2009	up to third level	multiclass	single	multiple data sources
Chen et al.^24^	Apr, 2012	up to first level	binary	multiple	multiple data sources
NetPredATC^12^	Apr, 2013	up to fourth level	binary	single	multiple data sources
iSEA^25^	Jul, 2013	up to second level	binary	single	chemical structure
Chen et al.^26^	Apr, 2014	up to first level	multiclass	multiple	multiple data sources
SuperPred 2.0 ^27^	May, 2014	up to fourth level	multiclass	single	chemical structure
SPACE^28^	Jan, 2015	up to fourth level	binary	multiple	multiple data sources
dD-Hybrid^29^	Oct, 2015	up to fourth level	binary	multiple	multiple data sources
iATC-mISF^30^	Oct, 2016	up to first level	multiclass	multiple	multiple data sources
iATC-mHyb^31^	Apr, 2017	up to first level	multiclass	multiple	multiple data sources
EnsLIFT^32^	Apr, 2017	up to first level	multiclass	multiple	multiple data sources
Tiered Learning^33^	Jun, 2017	up to fourth level	multiclass	single	chemical structure, multiple data sources
Lumini and Nanni^13^	Sep, 2018	up to first level	multiclass	multiple	multiple data sources
SOM-based^34^	Sep, 2018	up to first level	multiclass	single	multiple data sources
ATC-NLSP^35^	Sep, 2019	up to first level	multiclass	multiple	multiple data sources
FUS1, FUS2 and FUS3 ^36^	Sep, 2019	up to first level	multiclass	multiple	multiple data sources
iATC-NRAKEL^15^	Oct, 2019	up to first level	multiclass	multiple	multiple data sources
iATC-FRAKEL^16^	Mar, 2020	up to first level	multiclass	multiple	multiple data sources
STD-ACT^37^	Mar, 2020	up to fourth level	multiclass	multiple	multiple data sources
iATC_Deep-mISF^38^	May, 2020	up to first level	multiclass	multiple	multiple data sources
LHD-based^39^	May, 2020	up to first level	multiclass	multiple	multiple data sources
CGATCPred^17^	Mar, 2021	up to first level	multiclass	multiple	multiple data sources
RNPredATC^40^	Jun, 2021	up to fourth level	binary	multiple	multiple data sources
DeepATC^18^	Jul, 2021	up to first level	multiclass	multiple	multiple data sources
MLSMOTE^14^	Dec, 2021	up to first level	multiclass	multiple	chemical structure
EnsATC^41^	Mar, 2022	up to first level	multiclass	multiple	multiple data sources
SuperPred 3.0 ^42^	May, 2022	up to fourth level	multiclass	multiple	chemical structure
DACPGTN^43^	Jun, 2022	up to first level	multiclass	multiple	multiple data sources
ATC-CNN^19^	Jul, 2022	up to first level	multiclass	multiple	chemical structure
PDATC-NCPMKL^22^	Dec, 2023	up to fourth level	binary	multiple	multiple data sources
PDATC-NCPMKL-2 ^23^	Mar, 2025	up to fourth level	binary	multiple	multiple data sources
GraphATC^10^	Apr, 2025	up to second level	multiclass	multiple	chemical structure
FACT^20^	Jul, 2025	up to fourth level	binary	multiple	multiple data sources

Our method is a multiclass, multilabel, and multimodal approach that can predict ATC codes up to the fourth level using a sequence-to-sequence generative approach, which, to the best of our knowledge, has not been proposed before. Our approach is based exclusively on the chemical structure of the compounds; thus, no external data are required, and it can be used both for repurposing scenarios and for the annotation of new drugs. Furthermore, this provides the first approach that aims to automatically predict the number of ATC codes that can be reliably assigned for each compound.

## Materials and Methods

This section describes the data set collection process, including how different data sources were integrated. We then detail the sequence-to-sequence prediction methods and their meta-model that are proposed in this work, as well as a brief description of the baseline methods.

### Data Sets

Our data set was constructed by integrating the following databases, requiring that each drug entry include key fields such as the generic name, ATC code, molecule type (e.g., small molecule label or molecular weight), and SMILES:

(1) DrugBank. Accession numbers for 2,588 FDA-approved drug structures were obtained from the DrugBank database^44^ (release January 2023). Using these accession numbers, an in-house script was applied to retrieve relevant information for each compound.
(2) ZINC15. Accession numbers for 1615 compounds corresponding to FDA-approved drugs were downloaded from the ZINC15 database.^45^ An in-house script was employed to extract key data for each compound.
(3) ChEMBL. The ChEMBL dump (version 34)^46^ in SQLite format (v3.26.0) was used for data retrieval and analysis through SQL queries.
(4) KEGG (Kyoto Encyclopedia of Genes and Genomes). KEGG IDs from the “New Drug Approvals in Japan” database on the KEGG website were used,^47^ with 860 compounds available at the time of query (December 2024). Available fields included: Date, KEGG ID, ATC, JTC (Japanese Therapeutic Category According to List of Approved Products by PMDA), Active Ingredient, Drug Name, Company, and Remark. For this study, KEGG ID, ATC, and Drug Name were retained. ChEBI IDs were used to obtain SMILES strings via a script.

Molecular structures were transformed using computational chemistry tools, including Open Babel^48^ and RDKit.^49^ For the ZINC and KEGG databases, molecular weights were retrieved, and compounds with *MW* ≤ 900 Da were classified as small molecules, as defined by DrugBank.^44^ Primarily, charge neutralization and standardization were carried out with the MolStandardize module in RDKit. These procedures removed monatomic counterions, neutralized molecular charges, and corrected ambiguous or nonstandard chemical representations (see the Supporting Information). The resulting neutral fragments were converted into canonical SMILES strings. The canonical SMILES column was then used to merge the five databases into a unified and standardized molecular data set.

Additional filters were applied, including the exclusion of compounds classified under first-level ATC code “V” (except V03AX) and the removal of multicomponent systems. Compounds lacking SMILES or ATC codes, as well as counterions or salts that did not contribute to the pharmacological activity, were excluded. These structures can be found in the Supporting Information.

Following this procedure, 1868 molecules were retained from DrugBank, 1152 from ZINC15, 2553 from ChEMBL, and 235 from KEGG, respectively. After removing duplicates, the training data set comprised 3203 molecules. Note that the integration of compounds into a unified database is constrained by differences in the isomeric and tautomeric representations adopted in each of the source databases. Statistics on the distribution of the ATC codes in our data set are provided in the Supporting Information.

In addition to the SMILES representation of molecules, an alternative representation that is also based on the chemical structure was used. Molecular descriptors were calculated from their SMILES strings using Mordred.^50,51^ After the removal of highly correlated descriptors and those that exhibit constant or null values, a total of 1136 molecular descriptors were obtained for each molecule, comprising one- and two-dimensional descriptors.

### Models

In this article, we propose the use of different well-established neural architectures that are designed to leverage sequence-based learning. Our proposal uses only structural data from molecules, starting from their SMILES sequences, to predict the associated ATC codes. For the sake of comparison, we experimented with some typical baseline models, which are briefly explained below.

#### Baselines

Although in the related work section we presented a large number of studies that aim to predict ATC codes, our proposed methods differ from most of the existing approaches in several critical aspects, which makes them not directly comparable. In particular, most methods use input data from multiple sources, whereas our approach uses information only derived from the chemical structure of the molecules in order to ensure applicability to novel or poorly characterized compounds. Among the few studies that rely on chemical structure data, only a small subset propose multilabel and multiclass prediction strategies comparable to ours, some of which restrict predictions up to the first or second level of the ATC hierarchy. Furthermore, the analysis of the ATC code distribution in our data set (see the Supporting Information) shows that many compounds are annotated with multiple ATC codes, highlighting the complexity associated with the multilabel nature of this task and reinforcing the need for such models. For the few remaining methods that could have been used for comparison, the source code or data set used that would allow us to replicate the experiments is not available.

For the reasons stated above, we implemented several baselines for comparison with our proposed models. Each method outputs a prediction for each level of the ATC that is concatenated to form the four-level ATC code. The first baseline is a random classifier that, at each level of the ATC code, outputs its prediction by using the probability distribution of the ATC codes for that level.

Other methods were considered for comparison, namely, Random Forest (RF) and Feed-Forward Neural Networks (FFNN). Both were used in a hierarchical manner in which a model was trained for each level of the ATC codes. At the first level, the models were trained using the molecular descriptors information on the molecules. For the subsequent ATC levels, the models were trained using molecular descriptors and also information regarding the ATC codes for the preceding levels. At inference time, at each level, each ATC character is predicted by sampling from the probabilities output by the model. The chosen character is also fed to the model for inference at the next level. Random Forest was trained using Scikit-learn.^52^ Meanwhile, the feed-forward neural network was built using Keras,^53^ containing only one hidden layer with a sigmoid activation, using the Adam optimizer for minimizing a binary cross-entropy loss.

#### Proposed Models

Our proposed models were designed to leverage the string sequences in both SMILES and ATC codes by means of sequence-to-sequence architectures,^54^ which work as supervised encoder-decoder models. In other words, our models were trained to map SMILES sequences to ATC sequences up to the fourth level. We experimented with two deep learning architectures: a bidirectional recurrent neural network with long–short-term memory (LSTM)^7^ units and a transformer.^8^ The implementation of both methods was derived from the package pytorch_beam_search.^55^

While the original seq-to-seq LSTM implementation^56^ has both encoder and decoder as unidirectional LSTMs, ours uses a bidirectional LSTM (BiLSTM) in the encoder, whose forward and backward hidden states are properly combined and then passed to the unidirectional LSTM decoder to generate the ATC code. In the case of the transformer architecture, our implementation followed the standard model of Vaswani et al.^8^ Prior to the training of these models, a conventional character-wise tokenization of both SMILES and ATC strings was performed. At inference time, to retrieve the predicted sequences associated with the ATC codes up to the fourth level, a beam search algorithm was employed to generate multiple ATC codes.

A further improvement of the previous sequence-to-sequence algorithms was also proposed. Given that those models only used the SMILES information during training, we incorporated molecular descriptors to enrich drug representation and hence incorporate complementary information from another source of data, yet without involving external wet-lab information. This approach, known as multimodal learning, involves modifying the original architectures to exploit both representations. The encoded SMILES sequences were concatenated with the molecular descriptors and then passed through a fully connected layer. After that, the resulting fused representation was fed into the decoder, thereby enriching the learning process of these sequence methods (Figure 2).

**Figure 2:**
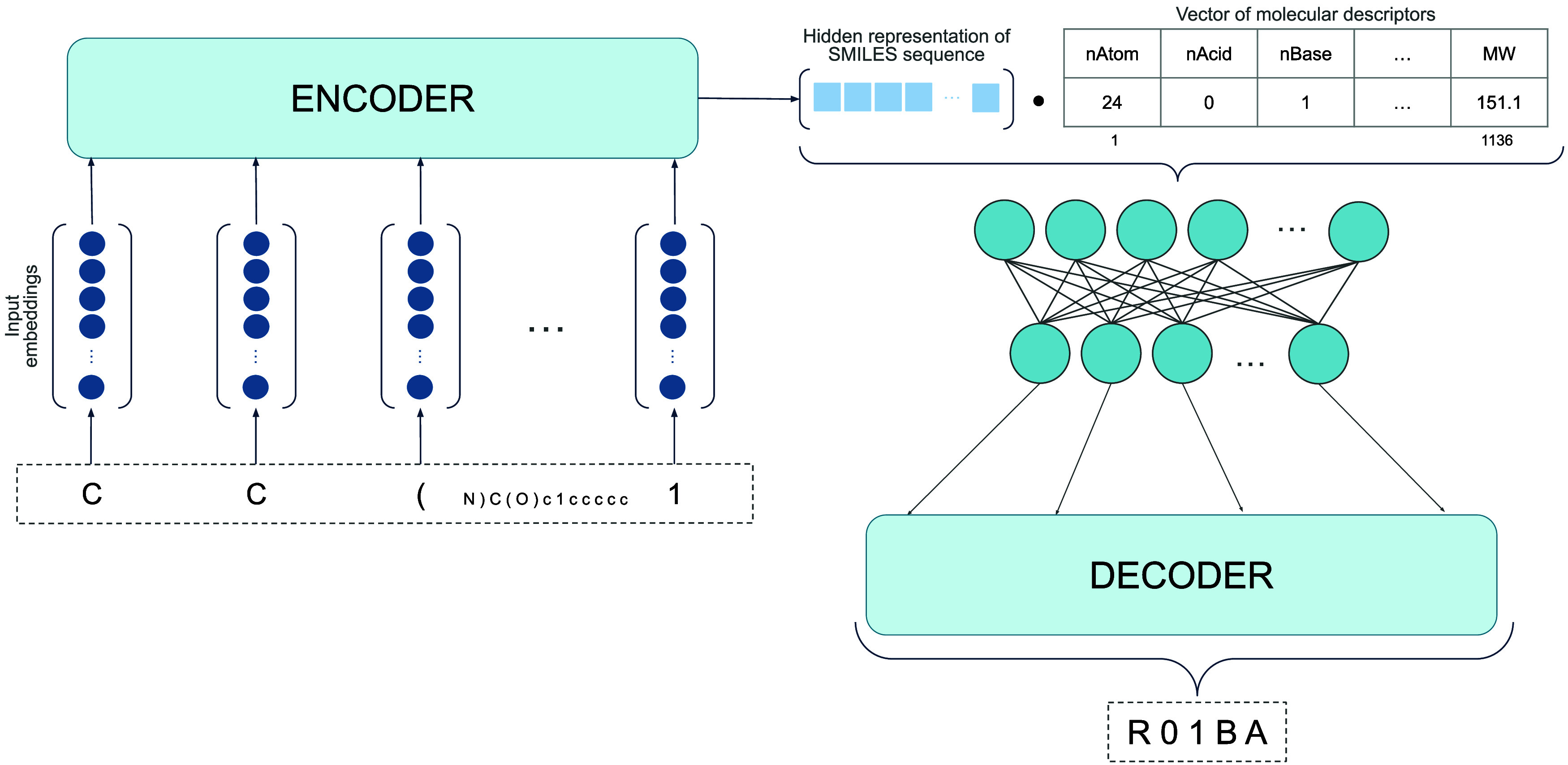
Schematic representation of the proposed multimodal sequence-to-sequence architecture at the inference time.

In order to account for drug repurposing, it is desirable that more than one ATC code be predicted per drug. This does not represent an issue for sequence-based generative models, where more than one output could be sampled based on different strategies (e.g., greedy, beam search). However, an important issue to address is how to determine the number of different ATC codes to be generated. Therefore, we proposed a novel meta-model to predict the number of ATC codes to be output. This model was implemented using a feed-forward neural network that contained two fully connected layers. The network input consisted of a vector of the ten highest probabilities associated with the generated ATC codes for each compound. Based on these probabilities, the model learned from the training set the appropriate number of ATC code predictions for each molecule in order to maximize a given metric, which in our case was the F1 score. This process is illustrated in Figure 3.

**Figure 3:**
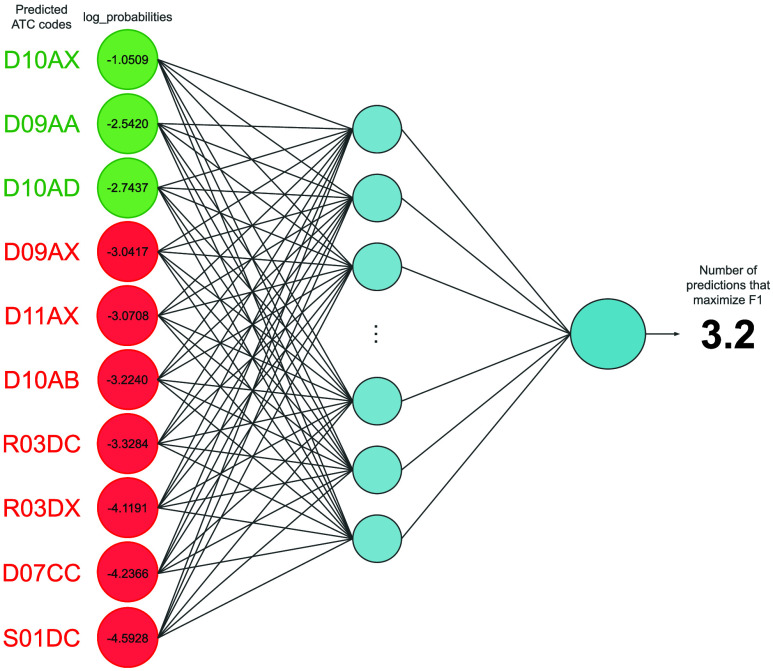
Schematic representation of the meta-model at training time. Green ATC codes represent the F1-optimal ATC codes that should be generated for a given molecule. The model should predict such a number based on the output log probabilities.

## Results

In this section, we first introduce the metrics used to evaluate the performance of our methods. Next, we outline the experimental design and report results by comparing the proposed models with the baselines under different scenarios.

### Performance Evaluation Metrics

We evaluated our models using two sets of metrics. The first set considered the four levels of the ATC codes and checked whether those codes had been exactly generated as in the annotated data set. For this set of metrics, we use the standard precision, recall, and F1 score, defined as in typical supervised machine learning settings. The second set of metrics was defined to better assess the methods in the context of our multilabel and multilevel problem. Due to the hierarchical nature of these codes (i.e., different ATC levels), we find it necessary to adapt the metrics for this type of task.

We redefined both precision and recall and named them L-Precision and L-Recall, to account for the correctness at different levels of the ATC codes, obtaining a measurement for each level. In order to evaluate the prediction of an ATC character at level *n*, we considered only those molecules for which at least one prediction was correct at the previous level (i.e., at level *n*–1). This is because if, for instance, at level 1 the predicted code is incorrect, since level 2 categorization depends on the previous level, then there is no point in assessing the correctness at the second level. In other words, a predicted ATC code for a certain compound is evaluated at level *n* only if that code was correct from level 1 to level *n*–1. Likewise, if none of the predicted ATC codes for a compound are valid predictions at level *n*, then this compound no longer contributes to evaluations at subsequent levels.

For each level of the ATC codes, precision is defined as the proportion of correctly predicted ATC codes to the number of predicted labels for that compound, averaged over the number of compounds that were evaluated (i.e., those that had at least one valid prediction at the previous level). Likewise, recall is defined as the proportion of correctly predicted ATC codes to the number of actual labels for that compound, averaged over the number of compounds that were evaluated at that level. These metrics allow one to determine whether certain levels are more difficult to learn than others without negatively impacting the prediction assessment when a mistake is made at a previous level. Formally, these metrics are defined as follows:

1 $$L‐Precision\left(C,n\right)=\frac{1}{\left|C^{n-1}\right|}\sum_{\alpha \in C^{n-1}\;}\frac{L‐Precision\left(\alpha ,n\right)}{\left|{Ŷ}_{\alpha }^{n}\right|}$$

2 $$L‐\mathrm{Precision}\left(\alpha ,n\right)=\sum_{ŷ\in {Ŷ}_{\alpha }^{n}\;}\left[\exists y\in Y_{\alpha }^{n}:\left(y=ŷ\right)\right]$$

3 $$L‐\mathrm{Recall}\left(C,n\right)=\frac{1}{\left|C^{n-1}\right|}\sum_{\alpha \in C^{n-1}\;}\frac{L‐\mathrm{Recall}\left(\alpha ,n\right)}{\left|Y_{\alpha }^{n}\right|}$$

4 $$L‐\mathrm{Recall}\left(\alpha ,n\right)=\sum_{y\in Y_{\alpha }^{n}\;}\left[\exists ŷ\in {Ŷ}_{\alpha }^{n}:\left(y=ŷ\right)\right]$$

where:

- *C*: set of compounds to assess prediction performance.
- *n*: ATC code level (i.e., 1 ≤ *n* ≤ 4).
- *C* ^*n*^: subset of compounds of *C* that have at least one correct ATC prediction at level *n*. We define *C* ^0^ = *C.*
- α: an arbitrary compound where we assess its prediction performance.
- *Ŷ*_α_^*n*^: for *n* = 1, this is the set of predicted ATC codes of molecule α at level 1. For *n* > 1, this is the set of predicted ATC codes of α at level *n* that were correctly predicted up to level *n*–1.
- *Y*_α_^*n*^: for *n* = 1, this is the set of actual ATC codes of molecule α at level 1. For *n* > 1, this is the set of actual ATC codes of α at level *n* that were correctly predicted up to level *n*–1.

In summary, we proposed two complementary sets of metrics to better characterize the performance of our models. The standard metrics are useful because they are easy to interpret, and they also assess the reliability of the models when generating the whole ATC codes up to level four. In contrast, the level-wise metrics are not standard in the literature, but they provide a more detailed view of the performance at each hierarchical level. These latter metrics offer deeper insight into the underlying process of ATC code generation up to level four and how performance changes as different levels are predicted.

### Experimental Design

To train and test our models, we conducted experiments in two different scenarios: one for annotating ATC codes to new drugs and another for drug repurposing. In the first scenario, we split the data set into training, validation, and test partitions maintaining the proportion of compounds with multiple ATC codes, while making sure that there is no repeated molecule in different data partitions. In contrast, in the second scenario, i.e., drug repurposing, we divided the data set according to the number of ATC codes assigned to each compound. All drugs with a single ATC code were assigned to the training partition. In the case of drugs with more than one ATC code, the set of codes was randomly split in half, with one-half assigned to the training set and the other to the test set. The validation set was randomly drawn from the training set, maintaining the proportion of compounds with multiple ATC codes. The same training, validation, and test partitions were applied to all models in both scenarios. This setting was repeated 10 times, using different random seeds, to account for the variability originated from the split of the data.

Following the definition of the methods and scenarios, we conducted a process to select optimal hyperparameter values. A set of hyperparameters was tuned for the random forest and neural network baseline models. For the BiLSTM-based models, the tuned hyperparameters were: encoder and decoder embedding dimension, number of layers and number of hidden units, dropout rate, weight decay, and learning rate. In the case of the Transformer-based models, the following hyperparameters were optimized: embedding dimension, feed-forward network dimension, number of encoder and decoder layers, number of attention heads, dropout rate, weight decay, and learning rate. A random-search-based tuning was performed for each of the six models, and the best combination that ensured a good balance between precision and recall—on a validation set not used for testing—was selected. Additional information on the hyperparameter tuning for the remaining methods can be found in our GitHub repository.

In the first scenario, where we predicted ATC codes for new drugs, we evaluated the models following the previously mentioned protocol. Results are reported when one ATC code is predicted per compound. Further results below will show how performance changes as we vary the number of predictions per compound. The performance metrics by level on the test set, averaged over ten independent train–test split repetitions, are presented in Table 2. These results show that sequence-to-sequence approaches achieve the best performance. Multimodal sequence-based approaches tend to perform the best for the first three levels, while for the fourth level, there is little difference with the SMILES-only sequence-based methods. When evaluated using the standard metrics based on the complete four-level ATC code, the results confirm that the multimodal sequence-based approaches perform best, as shown in Table 3. This indicates that incorporating molecular descriptors improves all metrics regardless of the type of underlying sequence-to-sequence architecture. This Table further highlights the performance differences between the baselines and proposed methods.

**Table 2:** ATC Codes Prediction Performance for New Drugs^a^

	L-precision				L-recall			
models	level 1	level 2	level 3	level 4	level 1	level 2	level 3	level 4
Random^b^	0.118 ± 0.015	0.177 ± 0.026	0.250 ± 0.062	0.087 ± 0.144	0.102 ± 0.013	0.170 ± 0.027	0.235 ± 0.061	0.087 ± 0.144
Random Forest^b^	0.407 ± 0.028	0.646 ± 0.035	0.738 ± 0.036	0.669 ± 0.029	0.366 ± 0.027	0.631 ± 0.035	0.703 ± 0.039	0.649 ± 0.027
Neural Network^b^	0.317 ± 0.010	0.493 ± 0.029	0.549 ± 0.053	0.358 ± 0.078	0.284 ± 0.011	0.485 ± 0.028	0.524 ± 0.055	0.334 ± 0.084
BiLSTM^b^	0.477 ± 0.027	0.772 ± 0.027	0.871 ± 0.026	**0. 786** ± **0. 053**	0.433 ± 0.025	0.753 ± 0.028	0.819 ± 0.027	0.757 ± 0.049
Transformer^b^	0.300 ± 0.054	0.575 ± 0.078	0.655 ± 0.173	0.558 ± 0.119	0.267 ± 0.049	0.565 ± 0.076	0.612 ± 0.168	0.531 ± 0.115
M.BiLSTM^b^	0.575 ± 0.023	**0. 828** ± **0. 031**	**0. 884** ± **0. 038**	0.783 ± 0.050	0.524 ± 0.019	0.811 ± 0.030	0.837 ± 0.034	0.750 ± 0.051
M.Transformer^b^	**0. 577** ± **0. 027**	0.825 ± 0.020	**0. 884** ± **0. 019**	0.721 ± 0.056	0.526 ± 0.023	0.806 ± 0.019	0.837 ± 0.017	0.691 ± 0.059
Meta-model	0.565 ± 0.022	0.814 ± 0.023	0.869 ± 0.037	0.751 ± 0.044	**0. 577** ± **0. 022**	**0. 822** ± **0. 027**	**0. 871** ± **0. 029**	**0. 786** ± **0. 040**

a Level-wise evaluation metrics are reported. M.BiLSTM and M.Transformer stand for the multimodal versions of the bidirectional LSTM and transformer, respectively. Bold values indicate the best mean performance for each metric.

b The number of predicted ATC codes is fixed to one.

**Table 3:** ATC Codes Prediction Performance for New Drugs^a^

models	precision	recall	F1
Random^b^	0.001 ± 0.001	0.000 ± 0.001	0.000 ± 0.001
Random Forest^b^	0.130 ± 0.014	0.107 ± 0.014	0.113 ± 0.014
Neural Network^b^	0.031 ± 0.007	0.023 ± 0.007	0.025 ± 0.007
BiLSTM^b^	0.252 ± 0.029	0.200 ± 0.025	0.214 ± 0.026
Transformer^b^	0.073 ± 0.040	0.053 ± 0.031	0.058 ± 0.033
M.BiLSTM^b^	**0. 332** ± **0. 047**	0.266 ± 0.039	0.283 ± 0.040
M.Transformer^b^	0.304 ± 0.036	0.242 ± 0.030	0.258 ± 0.032
Meta-model	0.308 ± 0.042	**0. 326** ± **0. 043**	**0. 298** ± **0. 040**

a Standard evaluation metrics are used to evaluate the prediction of complete ATC codes (up to the fourth level). M.BiLSTM and M.Transformer stand for the multimodal versions of the Bidirectional LSTM and Transformer, respectively. Bold values indicate the best mean performance for each metric.

b The number of predicted ATC codes is fixed to one.

In order to evaluate the meta-model in determining the number of ATC codes to generate per compound, we used our top-performing method as the base model, i.e., the multimodal BiLSTM. When training this meta-model, we generated ten different ATC codes for each compound in the training set. Based on the output log probabilities, the meta-model learned the number of ATC codes that maximizes the F1 score for each compound, which we then evaluated on a test subset. The meta-model generated one ATC code for 44.9% of the molecules in the test set, two ATC codes 53.1% of the times, and three ATC codes 2.0% of the times. The bottom row of Table 2 shows that, compared to predicting one ATC code, the meta-model achieves the highest recall without having a significant negative impact on precision. Furthermore, Table 3 confirms that using this adaptive strategy results in a higher F1 score, highlighting the advantages of not having to fix the number of ATC codes to be predicted in advance.

To analyze more thoroughly the impact of the number of ATC codes to be generated per compound, we evaluated each model when ranging from one to ten different ATC codes for each compound. The resulting F1 scores for each model are presented in Figure 4. This figure shows that the ranking of best performances among the models remains consistent regardless of the number of ATC codes generated per compound. The decision to predict a single ATC code when evaluating the models in both scenarios was made based on the results of this figure, which shows that fixing the number of predictions to one maximizes the F1 score. Additionally, Figure 4 presents the F1 score obtained by adapting the number of ATC codes generated for each compound with the predictions made by the meta-model (dashed line). It can be appreciated that this adaptive behavior renders a better F1 score than any of the fixed strategies, making it more suitable for a realistic setting when there is no certainty about the number of ATC codes to predict for a given compound.

**Figure 4:**
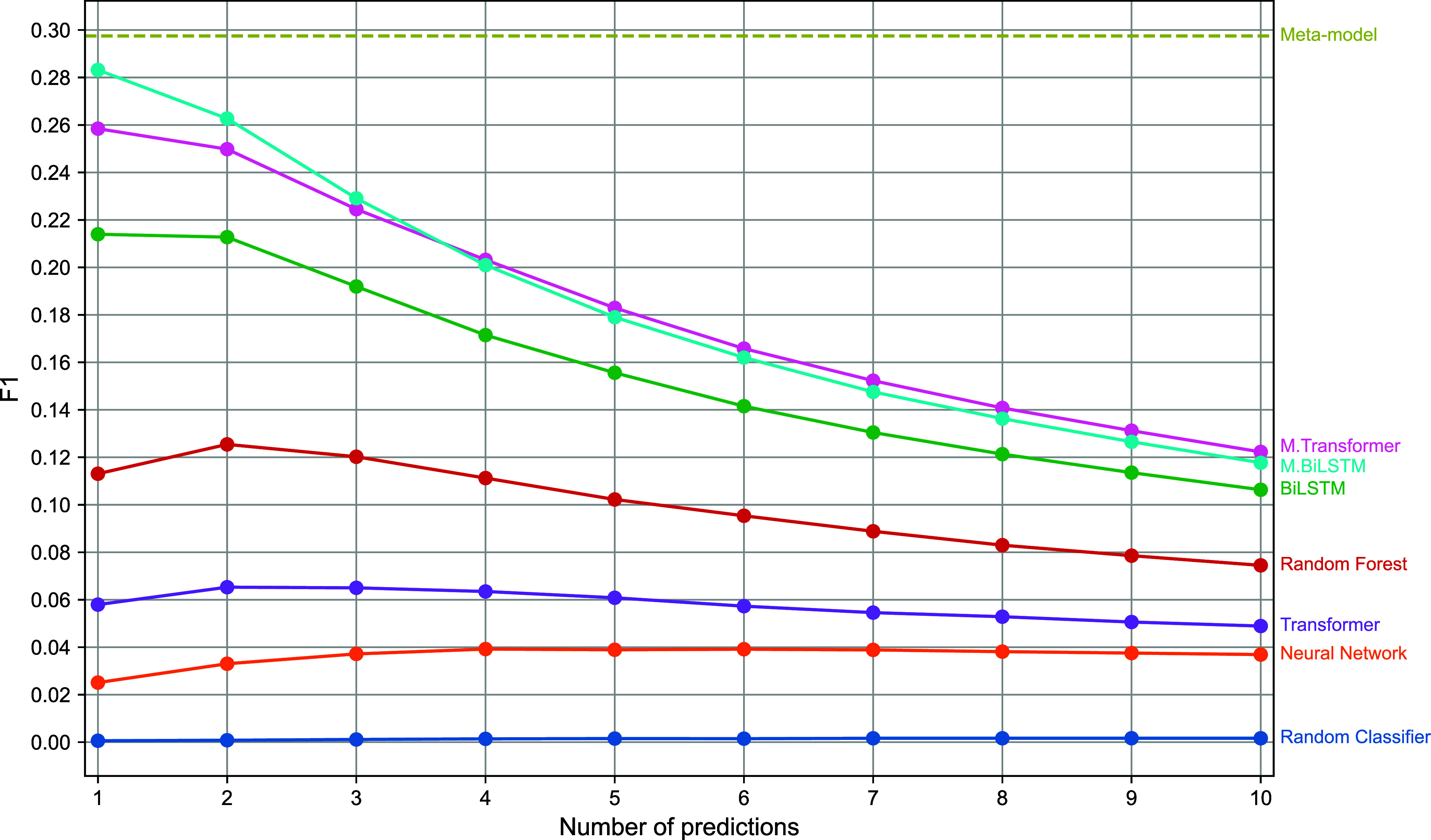
Comparison between the meta-model (dashed line) and the evaluated methods when varying the number of generated ATC codes per compound in terms of F1 score. The comparison shows that using the meta-model to adapt the number of ATC codes to be generated for each compound yields superior F1 score compared to fixing in advance the number of ATC codes to be generated.

For the second scenario, which aims to predict additional ATC codes for drugs that were already observed during training, we evaluated the models using the same protocol as that in the first scenario. The mean and standard deviation of the evaluation metrics on the test set are reported in Tables 4 and 5. As in the first scenario, the number of generated ATC codes was fixed to one, since this configuration yields the highest F1 score on this data set. An overall inspection of the results in Table 4 shows that the proposed multimodal sequence-to-sequence models achieve the best performance, substantially outperforming the considered baseline methods. More specifically, Table 5 clearly shows that the multimodal BiLSTM reaches the highest performance, whereas the multimodal Transformer achieves the second highest values. The results also show that as expected, the performance metrics for the drug repurposing scenario are slightly lower than those observed for the first scenario. However, the obtained performance remains encouraging, given the inherent difficulty of the task and the potential impact of drug repurposing efforts. In this setting, the challenge arises from the need for the model to generate novel therapeutic indications for a given compound, in addition to the already known ones. In addition, the data set is likely to contain incomplete annotations of all possible ATC codes associated with each compound. Therefore, predicting an ATC code that is not currently annotated for a compound does not necessarily correspond to a false positive. In this scenario, predictive models are not intended to provide definitive therapeutic assignments but rather to prioritize plausible new indications. Consequently, even moderate predictive performance can therefore be highly valuable if it helps narrow the experimental search space and generate biologically plausible hypotheses for further validation.

**Table 4:** ATC Codes Prediction Performance for Repurposing^a^

	L-precision				L-recall			
models	level 1	level 2	level 3	level 4	level 1	level 2	level 3	level 4
Random^b^	0.097 ± 0.008	0.155 ± 0.046	0.295 ± 0.136	0.082 ± 0.136	0.087 ± 0.008	0.151 ± 0.045	0.292 ± 0.139	0.082 ± 0.136
Random Forest^b^	0.462 ± 0.019	0.591 ± 0.023	0.553 ± 0.038	0.584 ± 0.049	0.433 ± 0.019	0.583 ± 0.023	0.539 ± 0.037	0.575 ± 0.050
Neural Network^b^	0.291 ± 0.027	0.502 ± 0.033	0.501 ± 0.070	0.319 ± 0.082	0.269 ± 0.026	0.495 ± 0.033	0.485 ± 0.066	0.308 ± 0.081
BiLSTM^b^	0.470 ± 0.052	0.781 ± 0.027	0.785 ± 0.023	**0. 744** ± **0. 033**	0.437 ± 0.052	0.770 ± 0.025	0.762 ± 0.022	**0. 731** ± **0. 032**
Transformer^b^	0.354 ± 0.043	0.687 ± 0.046	0.679 ± 0.059	0.606 ± 0.085	0.327 ± 0.042	0.677 ± 0.046	0.649 ± 0.061	0.592 ± 0.080
M.BiLSTM^b^	0.569 ± 0.021	**0. 836** ± **0. 021**	**0. 799** ± **0. 030**	0.737 ± 0.041	0.531 ± 0.018	0.822 ± 0.020	0.776 ± 0.028	0.724 ± 0.041
M.Transformer^b^	**0. 578** ± **0. 022**	0.813 ± 0.024	0.772 ± 0.035	0.728 ± 0.043	0.541 ± 0.023	0.802 ± 0.023	0.751 ± 0.035	0.717 ± 0.043
Meta-model	0.567 ± 0.020	0.829 ± 0.022	0.795 ± 0.029	0.725 ± 0.040	**0. 567** ± **0. 017**	**0. 828** ± **0. 019**	**0. 793** ± **0. 023**	0.730 ± 0.036

a Level-wise evaluation metrics are reported. M.BiLSTM and M.Transformer stand for the multimodal versions of the Bidirectional LSTM and Transformer, respectively. Bold values indicate the best mean performance for each metric.

b The number of predicted ATC codes is fixed to one.

**Table 5:** ATC Codes Prediction Performance for Repurposing^a^

models	precision	recall	F1
Random^b^	0.001 ± 0.001	0.000 ± 0.001	0.000 ± 0.001
Random Forest^b^	0.088 ± 0.012	0.079 ± 0.011	0.082 ± 0.011
Neural Network^b^	0.023 ± 0.006	0.019 ± 0.005	0.020 ± 0.005
BiLSTM^b^	0.214 ± 0.024	0.186 ± 0.024	0.194 ± 0.024
Transformer^b^	0.104 ± 0.032	0.086 ± 0.027	0.091 ± 0.029
M.BiLSTM^b^	**0. 280** ± **0. 022**	0.245 ± 0.020	0.255 ± 0.021
M.Transformer^b^	0.264 ± 0.029	0.233 ± 0.028	0.242 ± 0.028
Meta-model	0.276 ± 0.022	**0. 271** ± **0. 021**	**0. 263** ± **0. 021**

a Standard evaluation metrics are reported to evaluate the prediction of complete ATC codes (up to the fourth level). M.BiLSTM and M.Transformer stand for the multimodal versions of the Bidirectional LSTM and Transformer, respectively. Bold values indicate the best mean performance for each metric.

b The number of predicted ATC codes is fixed to one.

Finally, following the same protocol as in the first scenario, we trained and tested the meta-model using the multimodal BiLSTM as the base model, which generated one ATC code for 71.2% of the molecules in the test set and two ATC codes for the remaining 28.8% of the molecules. Based on the performance metrics shown in Tables 4 and 5, we can conclude that using this adaptive prediction strategy is also beneficial in the drug repurposing scenario. The meta-model achieves the highest recall and F1 scores, while precision is not significantly affected as it is slightly lower compared to the best values. Additionally, the comparison between the meta-model and the evaluated methods when the number of predicted ATC codes per compound varies from one to ten shows similar patterns to the ones in the first scenario. This figure can be found in the Supporting Information.

## Conclusions

In this work, we addressed the problem of automatically predicting ATC codes for compounds using a novel approach while also considering several design decisions. First, we designed our method to use only chemical structure information for the compounds so that ATC codes can be predicted not only for repurposing tasks but also for annotating new compounds. Another relevant design decision was to approach our prediction problem as multiclass and multilabel, thus modeling the inherent nature of the problem as closely as possible, where multiple multilevel ATC codes can exist for a single compound.

The proposal of sequence-to-sequence architectures that map SMILES to ATC codes was shown to be effective. Specifically, we worked with two architectures: BiLSTM and Transformers. We also tested whether a multimodal version of the sequence-based approaches that incorporates molecular descriptors into the representation would improve the prediction performance. The experiments showed an improvement in most metrics compared to the other competing models tested in this work. This higher performance was consistently observed for both scenarios (predictions of new drugs and drug repurposing) and for most hierarchical levels of the ATC code.

Finally, given the capacity of generating multiple ATC codes per compound and the potential of a drug to have more than one ATC code, we decided that it was relevant to provide a prediction method to determine the number of labels to reliably accept as outputs. To this end, we trained a model that infers the optimal number of ATC labels that the sequence-based model should generate as output. The results backed up this strategy as it allowed one to infer the number of ATC predictions to generate per compound more accurately in terms of F1 score, as opposed to keeping this number constant regardless of the method’s output probability during sequence generation. This allows our method to be reliably applied in a real setting. Future work could explore approaches to improve the interpretability of ATC prediction models. We expect that the prediction performance of our approach could further improve if larger data sets become available. The publication of our code and data set makes it possible for the research community to build more robust methods on top of the presented contributions.

## Supplementary Material



## Data Availability

The complete data set, together with the corresponding data partitions and the source code used to train and validate the models, is publicly available through this GitHub repository: https://github.com/TrinidadCrozes/Multimodal-seq2seq-ATC-generator.

## Notes

Further information about collection and filtering of the data set, statistics on the ATC code distribution, metric formulas, and some examples of their use, as well as a comparison between the meta-model and the evaluated methods in the drug repurposing scenario (PDF)
