# Supplementary material for: A Multimodal Sequence-to-Sequence Model for Automatic Assignment of ATC Codes in Drug Discovery and Repurposing
Source: J Chem Inf Model. 2026 Apr 14;66(8):4472–83. doi: 10.1021/acs.jcim.6c00118 (PMC13126624; doi:10.1021/acs.jcim.6c00118)
Supplement: Supplementary file 1 [file ci6c00118_si_001.pdf]

Supporting Information
A Multimodal Sequence-to-Sequence Model for Automatic Assignment of ATC Codes in Drug Discovery and Repurposing

Trinidad Crozes,[†,‡] Eugenia Ulzurrun,[¶] Juan A. Páez,[§] Nuria E. Campillo,[*,¶] Axel J. Soto,[†,‡] and Ignacio Ponzoni[*,†,‡]

†*Institute for Computer Science and Engineering, UNS-CONICET, 8000, Bahía Blanca, Argentina*

‡*Department of Computer Science and Engineering, Universidad Nacional del Sur, 8000, Bahía Blanca, Argentina.*

¶*Centro de Investigaciones Biológicas Margarita Salas (CIB Margarita Salas-CSIC). C/Ramiro de Maeztu, 9, 28040 Madrid, Spain.*

§*Instituto de Química Médica (IQM-CSIC). C/Juan de la Cierva, 3, 28006 Madrid, Spain.*

E-mail: nuria.campillo@csic.es; ip@cs.uns.edu.ar

Contents

Excluded structures (not contributing to pharmacological activity) **S3**

Statistics on the distribution of ATC codes in our dataset **S3**

Formulae for our metrics **S4**

Usage example S5

Experimental Design **S14**

Excluded structures (not contributing to pharmacological activity)

`NCC(=O)O, O=S(=O)(O)O, O=C(O)CN(CCN(CC(=O)O)CC(=O)O)CC(=O)O, O=C(O)O, O=C(O)CC(O)(CC(=O)O)C(=O)O, CC(O)C(=O)O, O=P(O)(O)O, O=[N+]([O-])O, O=C(O)[C@H](O)[C@@H](O)[C@H](O)[C@H](O)CO, II, [N]=O, S=[Se]=S, [N-]=[N+]=O, S1SSSSSSS1, OO, Ocl, ClC(Cl)Cl, O=O, N#N, O=C=O, O=[Zn], Cl[Al](Cl)Cl, F[Sn]F, Cl[201Tl], C[S+](C)[O-], CC(=O)CCC(=O)O, CCCl, O=[Mg], OCCO, C=COC=C, C=CCCCCCCCCC(=O)O, O=[As]O[As]=O, CCO, O[Al](O)O, ClC=C(Cl)Cl, O=[Si](O)O, CCCO, O=P(O)(O)F, CCOCC, N=C=N,CC(=O)CC(=O)O, NC(N)=O, NCCO, OC(CI)CI, CC(C)O, O=[Se](O)O, O=[Al]O[Al]=O, IC(I)I, OB(O)O, CC(=O)O.`

Statistics on the distribution of ATC codes in our dataset

Table S1: This table shows the number of compounds-ATC pairs per level as well as the number of different ATC code categories per level.

3203 compounds		
Level	# (SMILES, ATC)	# Categories of ATC codes
1	3795	14
2	3973	81
3	4253	211
4	4510	665

Table S2: Number of annotated ATC compounds in our dataset. As the table shows most compounds (i.e. 2442 compounds) have only one annotated ATC code, while more than 700 have more than one annotated ATC code.

**Level-fourth ATC codes per compound**	Number of compounds
**1**	2442
**2**	511
**3**	128
**4**	58
**5**	30
**6**	11
**7**	9
**8**	6
**9**	3
**11**	1
**15**	1
**16**	2
**18**	1

Formulae for our metrics

$$\textit{L-Precision}(C, n) = \frac{1}{|\mathrm{C}^{\mathrm{n}-1}|} \sum_{\alpha \in C^{n-1}} \frac{\textit{L-Precision}(\alpha, n)}{|\hat{Y}_\alpha^n|} \tag{1}$$

$$\textit{L-Precision}(\alpha, n) = \sum_{\hat{y} \in \hat{Y}_\alpha^n} [\exists y \in Y_\alpha^n : (y = \hat{y})] \tag{2}$$

$$\textit{L-Recall}(C, n) = \frac{1}{|\mathrm{C}^{\mathrm{n}-1}|} \sum_{\alpha \in C^{n-1}} \frac{\textit{L-Recall}(\alpha, n)}{|Y_\alpha^n|} \tag{3}$$

$$\textit{L-Recall}(\alpha, n) = \sum_{y \in Y_\alpha^n} [\exists \hat{y} \in \hat{Y}_\alpha^n : (y = \hat{y})] \tag{4}$$

where:

- C: set of compounds to assess prediction performance.
- n: ATC code level (i.e., $1 \leq n \leq 4$).
- C^n: subset of compounds of C that have at least one correct ATC prediction at level n. We define $C^0 = C$.

- α: an arbitrary compound where we assess its prediction performance.
- $\hat{Y}_{\alpha}^{n}$: for $n = 1$, this is the set of predicted ATC codes of molecule α at level 1. For $n > 1$, this is the set of predicted ATC codes of α at level n that were correctly predicted up to level $n - 1$.
- Y_{α}^{n}: for $n = 1$, this is the set of actual ATC codes of molecule α at level 1. For $n > 1$, this is the set of actual ATC codes of α at level n that were correctly predicted up to level $n - 1$.

Usage example

Set of drugs C = $\{Drug_a$, $Drug_b$, $Drug_c$, $Drug_d$, $Drug_e$, $Drug_f$, $Drug_g$, $Drug_h\}$

- $Drug_a$

  True codes: [S01AA, S02AA, D06AX, A07AA, J04AB]

  Predicted codes: [J01AA, J01FA, J01CA]

  - **Level 1:**

    $Y_{Drug_a}^{1} = [S, S, D, A, J]$

    $\hat{Y}_{Drug_a}^{1} = [J, J, J]$

    $\textbf{L-Precision}(\mathbf{Drug_a}, \mathbf{1}) = 1 + 1 + 1 = \mathbf{3}$

    $\textbf{L-Recall}(\mathbf{Drug_a}, \mathbf{1}) = 0 + 0 + 0 + 0 + 1 = \mathbf{1}$

  - **Level 2:**

    $Y_{Drug_a}^{2} = [J04]$

    $\hat{Y}_{Drug_a}^{2} = [J01, J01, J01]$

    $\textbf{L-Precision}(\mathbf{Drug_a}, \mathbf{2}) = 0 + 0 + 0 = \mathbf{0}$

    $\textbf{L-Recall}(\mathbf{Drug_a}, \mathbf{2}) = \mathbf{0}$

  - **Level 3:**

    $Y_{Drug_a}^{3} = []$

$\hat{Y}^{3}_{Drug_a} = []$

$\mathbf{L\text{-}Precision(Drug_a, 3) = 0}$

$\mathbf{L\text{-}Recall(Drug_a, 3) = 0}$

- **Level 4:**

  $Y^{4}_{Drug_a} = []$

  $\hat{Y}^{4}_{Drug_a} = []$

  $\mathbf{L\text{-}Precision(Drug_a, 4) = 0}$

  $\mathbf{L\text{-}Recall(Drug_a, 4) = 0}$

Standard metrics considering ATC codes up to level 4:

$Precision = \frac{0}{0+3} = 0$

$Recall = \frac{0}{0+5} = 0$

$F1 = 2 \times \frac{0 \times 0}{0+0} = 0$

- $Drug_b$

  True codes: [L01BA]

  Predicted codes: [J01DC, L01XX, J01DB]

  - **Level 1:**

    $Y^{1}_{Drug_b} = [L]$

    $\hat{Y}^{1}_{Drug_b} = [J, L, J]$

    $\mathbf{L\text{-}Precision(Drug_b, 1)} = 0 + 1 + 0 = \mathbf{1}$

    $\mathbf{L\text{-}Recall(Drug_b, 1)} = 1 = \mathbf{1}$

  - **Level 2:**

    $Y^{2}_{Drug_b} = [L01]$

    $\hat{Y}^{2}_{Drug_b} = [L01]$

    $\mathbf{L\text{-}Precision(Drug_b, 2)} = 1 = \mathbf{1}$

    $\mathbf{L\text{-}Recall(Drug_b, 2)} = 1 = \mathbf{1}$

- **Level 3:**

  $Y^3_{Drug_b} = [L01B]$

  $\hat{Y}^3_{Drug_b} = [L01X]$

  $\mathbf{L\text{-}Precision(Drug_b, 3)} = 0 = \mathbf{0}$

  $\mathbf{L\text{-}Recall(Drug_b, 3)} = 0 = \mathbf{0}$

- **Level 4:**

  $Y^4_{Drug_b} = []$

  $\hat{Y}^4_{Drug_b} = []$

  $\mathbf{L\text{-}Precision(Drug_b, 4)} = \mathbf{0}$

  $\mathbf{L\text{-}Recall(Drug_b, 4)} = \mathbf{0}$

Standard metrics considering ATC codes up to level 4:

$Precision = \frac{0}{0+3} = 0$

$Recall = \frac{0}{0+1} = 0$

$F1 = 2 \times \frac{0\times0}{0+0} = 0$

- $Drug_c$ True codes: [N06BX]

  Predicted codes: [N05CD, N06BA, S01ED]

  - **Level 1:**

    $Y^1_{Drug_c} = [N]$

    $\hat{Y}^1_{Drug_c} = [N, N, S]$

    $\mathbf{L\text{-}Precision(Drug_c, 1)} = 1 + 1 + 0 = \mathbf{2}$

    $\mathbf{L\text{-}Recall(Drug_c, 1)} = 1 = \mathbf{1}$

  - **Level 2:**

    $Y^2_{Drug_c} = [N06]$

    $\hat{Y}^2_{Drug_c} = [N05, N06]$

    $\mathbf{L\text{-}Precision(Drug_c, 2)} = 0 + 1 = \mathbf{1}$

    $\mathbf{L\text{-}Recall(Drug_c, 2)} = 1 = \mathbf{1}$

- **Level 3:**

  $Y^3_{Drug_c} = [N06B]$

  $\hat{Y}^3_{Drug_c} = [N06B]$

  $\textbf{L-Precision}(\mathbf{Drug_c}, \mathbf{3}) = 1 = \mathbf{1}$

  $\textbf{L-Recall}(\mathbf{Drug_c}, \mathbf{3}) = 1 = \mathbf{1}$

- **Level 4:**

  $Y^4_{Drug_c} = [N06BX]$

  $\hat{Y}^4_{Drug_c} = [N06BA]$

  $\textbf{L-Precision}(\mathbf{Drug_c}, \mathbf{4}) = \mathbf{0}$

  $\textbf{L-Recall}(\mathbf{Drug_c}, \mathbf{4}) = \mathbf{0}$

Standard metrics considering ATC codes up to level 4:

$Precision = \frac{0}{0+3} = 0$

$Recall = \frac{0}{0+1} = 0$

$F1 = 2 \times \frac{0 \times 0}{0+0} = 0$

- $Drug_d$

  True codes: [L01AD]

  Predicted codes: [J05AB, L01AC, L01AB]

  - **Level 1:**

    $Y^1_{Drug_d} = [L]$

    $\hat{Y}^1_{Drug_d} = [J, L, L]$

    $\textbf{L-Precision}(\mathbf{Drug_d}, \mathbf{1}) = 0 + 1 + 1 = \mathbf{2}$

    $\textbf{L-Recall}(\mathbf{Drug_d}, \mathbf{1}) = 1 = \mathbf{1}$

  - **Level 2:**

    $Y^2_{Drug_d} = [L01]$

    $\hat{Y}^2_{Drug_d} = [L01, L01]$

    $\textbf{L-Precision}(\mathbf{Drug_d}, \mathbf{2}) = 1 + 1 = \mathbf{2}$

$\textbf{L-Recall}(\textbf{Drug}_\textbf{d}, \textbf{2}) = 1 = \textbf{1}$

- **Level 3:**

  $Y^3_{Drug_d} = [L01A]$

  $\hat{Y}^3_{Drug_d} = [L01A, L01A]$

  $\textbf{L-Precision}(\textbf{Drug}_\textbf{d}, \textbf{3}) = 1 + 1 = \textbf{2}$

  $\textbf{L-Recall}(\textbf{Drug}_\textbf{d}, \textbf{3}) = 1 = \textbf{1}$

- **Level 4:**

  $Y^4_{Drug_d} = [L01AD]$

  $\hat{Y}^4_{drug_d} = [L01AC, L01AB]$

  $\textbf{L-Precision}(\textbf{Drug}_\textbf{d}, \textbf{4}) = 0 + 0 = \textbf{0}$

  $\textbf{L-Recall}(\textbf{Drug}_\textbf{d}, \textbf{4}) = 0 = \textbf{0}$

Standard metrics considering ATC codes up to level 4:

$Precision = \frac{0}{0+3} = 0$

$Recall = \frac{0}{0+1} = 0$

$F1 = 2 \times \frac{0 \times 0}{0+0} = 0$

- $Drug_e$

  True codes: [C07BA, C07AA, C07CA]

  Predicted codes: [C07AB, N07BA, N07AB]

  - **Level 1:**

    $Y^1_{Drug_e} = [C, C, C]$

    $\hat{Y}^1_{Drug_e} = [C, N, N]$

    $\textbf{L-Precision}(\textbf{Drug}_\textbf{e}, \textbf{1}) = 1 + 0 + 0 = \textbf{1}$

    $\textbf{L-Recall}(\textbf{Drug}_\textbf{e}, \textbf{1}) = 1 + 1 + 1 = \textbf{3}$

  - **Level 2:**

    $Y^2_{Drug_e} = [C07, C07, C07]$

    $\hat{Y}^2_{Drug_e} = [C07]$

$\mathbf{L\text{-}Precision(Drug_e, 2)} = 1 = \mathbf{1}$

$\mathbf{L\text{-}Recall(Drug_e, 2)} = 1 + 1 + 1 = \mathbf{3}$

- **Level 3:**

  $Y^3_{Drug_e} = [C07B, C07A, C07C]$

  $\hat{Y}^3_{Drug_e} = [C07A]$

  $\mathbf{L\text{-}Precision(Drug_e, 3)} = 1 = \mathbf{1}$

  $\mathbf{L\text{-}Recall(Drug_e, 3)} = 0 + 1 + 0 = \mathbf{1}$

- **Level 4:**

  $Y^4_{Drug_e} = [C07AA]$

  $\hat{Y}^4_{drug_e} = [C07AB]$

  $\mathbf{L\text{-}Precision(Drug_e, 4)} = 0 = \mathbf{0}$

  $\mathbf{L\text{-}Recall(Drug_e, 4)} = 0 = \mathbf{0}$

Standard metrics considering ATC codes up to level 4:

$Precision = \frac{0}{0+3} = 0$

$Recall = \frac{0}{0+3} = 0$

$F1 = 2 \times \frac{0 \times 0}{0+0} = 0$

- $Drug_f$

  True codes: [J05AR, J05AF]

  Predicted codes: [J05AR, J05AF, J05AE]

  - **Level 1:**

    $Y^1_{Drug_f} = [J, J]$

    $\hat{Y}^1_{Drug_f} = [J, J, J]$

    $\mathbf{L\text{-}Precision(Drug_f, 1)} = 1 + 1 + 1 = \mathbf{3}$

    $\mathbf{L\text{-}Recall(Drug_f, 1)} = 1 + 1 = \mathbf{2}$

  - **Level 2:**

    $Y^2_{Drug_f} = [J05, J05]$

$\hat{Y}^2_{Drug_f} = [J05, J05, J05]$

$\mathbf{L\text{-}Precision(Drug_f, 2)} = 1 + 1 + 1 = \mathbf{3}$

$\mathbf{L\text{-}Recall(Drug_f, 2)} = 1 + 1 = \mathbf{2}$

- **Level 3:**

  $Y^3_{Drug_f} = [J05A, J05A]$

  $\hat{Y}^3_{Drug_f} = [J05A, J05A, J05A]$

  $\mathbf{L\text{-}Precision(Drug_f, 3)} = 1 + 1 + 1 = \mathbf{3}$

  $\mathbf{L\text{-}Recall(Drug_f, 3)} = 1 + 1 = \mathbf{2}$

- **Level 4:**

  $Y^4_{Drug_f} = [J05AR, J05AF]$

  $\hat{Y}^4_{Drug_f} = [J05AR, J05AF, J05AE]$

  $\mathbf{L\text{-}Precision(Drug_f, 4)} = 1 + 1 + 0 = \mathbf{2}$

  $\mathbf{L\text{-}Recall(Drug_f, 4)} = 1 + 1 = \mathbf{2}$

Standard metrics considering ATC codes up to level 4:

$Precision = \frac{2}{2+1} = 0.667$

$Recall = \frac{2}{2+0} = 1$

$F1 = 2 \times \frac{0.667 \times 1}{0.667+1} = 0.8$

- $Drug_g$

  True codes: [V03AX]

  Predicted codes: [A06AX, R01AC, A06AD]

  - **Level 1:**

    $Y^1_{Drug_g} = [V]$

    $\hat{Y}^1_{Drug_g} = [A, R, A]$

    $\mathbf{L\text{-}Precision(Drug_g, 1)} = 0 + 0 + 0 = \mathbf{0}$

    $\mathbf{L\text{-}Recall(Drug_g, 1)} = 0 = \mathbf{0}$

- **Level 2:**

  $Y^2_{Drug_g} = []$

  $\hat{Y}^2_{Drug_g} = []$

  $\mathbf{L\text{-}Precision(Drug_g, 2) = 0}$

  $\mathbf{L\text{-}Recall(Drug_g, 2) = 0}$

- **Level 3:**

  $Y^3_{Drug_g} = []$

  $\hat{Y}^3_{Drug_g} = []$

  $\mathbf{L\text{-}Precision(Drug_g, 3) = 0}$

  $\mathbf{L\text{-}Recall(Drug_g, 3) = 0}$

- **Level 4:**

  $Y^4_{Drug_g} = []$

  $\hat{Y}^4_{Drug_g} = []$

  $\mathbf{L\text{-}Precision(Drug_g, 4) = 0}$

  $\mathbf{L\text{-}Recall(Drug_g, 4) = 0}$

Standard metrics considering ATC codes up to level 4:

$Precision = \frac{0}{0+3} = 0$

$Recall = \frac{0}{0+1} = 0$

$F1 = 2 \times \frac{0 \times 0}{0+0} = 0$

- $Drug_h$

  True codes: [S01FA, A03CB, A03BA]

  Predicted codes: [S01FA, S01FB, N06BA]

  - **Level 1:**

    $Y^1_{Drug_h} = [S, A, A]$

    $\hat{Y}^1_{Drug_h} = [S, S, N]$

    $\mathbf{L\text{-}Precision(Drug_h, 1)} = 1 + 1 + 0 = \mathbf{2}$

$\mathbf{L\text{-}Recall}(\mathbf{Drug_h}, \mathbf{1}) = 1 + 0 + 0 = \mathbf{1}$

- **Level 2:**

  $Y^2_{Drug_h} = [S01]$

  $\hat{Y}^2_{Drug_h} = [S01, S01]$

  $\mathbf{L\text{-}Precision}(\mathbf{Drug_h}, \mathbf{2}) = 1 + 1 = \mathbf{2}$

  $\mathbf{L\text{-}Recall}(\mathbf{Drug_h}, \mathbf{2}) = 1 = \mathbf{1}$

- **Level 3:**

  $Y^3_{Drug_h} = [S01F]$

  $\hat{Y}^3_{Drug_h} = [S01F, S01F]$

  $\mathbf{L\text{-}Precision}(\mathbf{Drug_h}, \mathbf{3}) = 1 + 1 = \mathbf{2}$

  $\mathbf{L\text{-}Recall}(\mathbf{Drug_h}, \mathbf{3}) = 1 = \mathbf{1}$

- **Level 4:**

  $Y^4_{Drug_h} = [S01FA]$

  $\hat{Y}^4_{Drug_h} = [S01FA, S01FB]$

  $\mathbf{L\text{-}Precision}(\mathbf{Drug_h}, 4) = 1 + 0 = \mathbf{1}$

  $\mathbf{L\text{-}Recall}(\mathbf{Drug_h}, 4) = 1 = \mathbf{1}$

Standard metrics considering ATC codes up to level 4:

$Precision = \frac{1}{1+2} = 0.333$

$Recall = \frac{1}{1+2} = 0.333$

$F1 = 2 \times \frac{0.333 \times 0.333}{0.333 + 0.333} = 0.333$

Proposed metrics per level:

$L\text{-}Precision(C, 1) = \frac{1}{8} \times (\frac{3}{3} + \frac{1}{3} + \frac{2}{3} + \frac{2}{3} + \frac{1}{3} + \frac{3}{3} + \frac{0}{3} + \frac{2}{3})$

$L\text{-}Precision(C, 1) = \frac{1}{8} \times \frac{14}{3} = 0.5833$

7 drugs had at least one valid prediction at level 1

$L\text{-}Precision(C, 2) = \frac{1}{7} \times (\frac{0}{3} + \frac{1}{1} + \frac{1}{2} + \frac{2}{2} + \frac{1}{1} + \frac{3}{3} + \frac{2}{2})$

$L\text{-}Precision(C, 2) = \frac{1}{7} \times \frac{11}{2} = 0.7857$

6 drugs had at least one valid prediction at level 2

$L\text{-}Precision(C, 3) = \frac{1}{6} \times (\frac{0}{1} + \frac{1}{1} + \frac{2}{2} + \frac{1}{1} + \frac{3}{3} + \frac{2}{2})$

$Precision_3 = \frac{1}{6} \times 5 = 0.8333$

5 drugs had at least one valid prediction at level 3

$L\text{-}Precision(C, 4) = \frac{1}{5} \times (\frac{0}{1} + \frac{0}{2} + \frac{0}{1} + \frac{2}{3} + \frac{1}{2})$

$L\text{-}Precision(C, 4) = \frac{1}{5} \times \frac{7}{6} = 0.2333$

$L\text{-}Recall(C, 1) = \frac{1}{8} \times (\frac{1}{5} + \frac{1}{1} + \frac{1}{1} + \frac{1}{1} + \frac{3}{3} + \frac{2}{2} + \frac{0}{1} + \frac{1}{3})$

$L\text{-}Recall(C, 1) = \frac{1}{8} \times \frac{83}{15} = 0.6917$

$L\text{-}Recall(C, 2) = \frac{1}{7} \times (\frac{0}{1} + \frac{1}{1} + \frac{1}{1} + \frac{1}{1} + \frac{3}{3} + \frac{2}{2} + \frac{1}{1})$

$L\text{-}Recall(C, 2) = \frac{1}{7} \times 6 = 0.8571$

$L\text{-}Recall(C, 3) = \frac{1}{6} \times (\frac{0}{1} + \frac{1}{1} + \frac{1}{1} + \frac{1}{3} + \frac{2}{2} + \frac{1}{1})$

$L\text{-}Recall(C, 3) = \frac{1}{6} \times \frac{13}{3} = 0.7222$

$L\text{-}Recall(C, 4) = \frac{1}{5} \times (\frac{0}{1} + \frac{0}{1} + \frac{0}{1} + \frac{2}{2} + \frac{1}{1})$

$L\text{-}Recall(C, 4) = \frac{1}{5} \times 2 = 0.4$

Standard metrics considering ATC codes up to level 4:

$Precision = \frac{1}{8} \times (0 + 0 + 0 + 0 + 0 + 0.667 + 0 + 0.333) = 0.125$

$Recall = \frac{1}{8} \times (0 + 0 + 0 + 0 + 0 + 1 + 0 + 0.333) = 0.167$

$F1 = \frac{1}{8} \times (0 + 0 + 0 + 0 + 0 + 0.8 + 0 + 0.333) = 0.142$

Experimental Design

For the drug repurposing scenario, Figure S1 shows that the ranking of performances among the models remains consistent regardless the number of ATC codes generated per compound. This figure also shows that the highest F1 score is achieved when the number of ATC codes generated for each compound is adapted according to the predictions made by the meta-

model (dashed line).

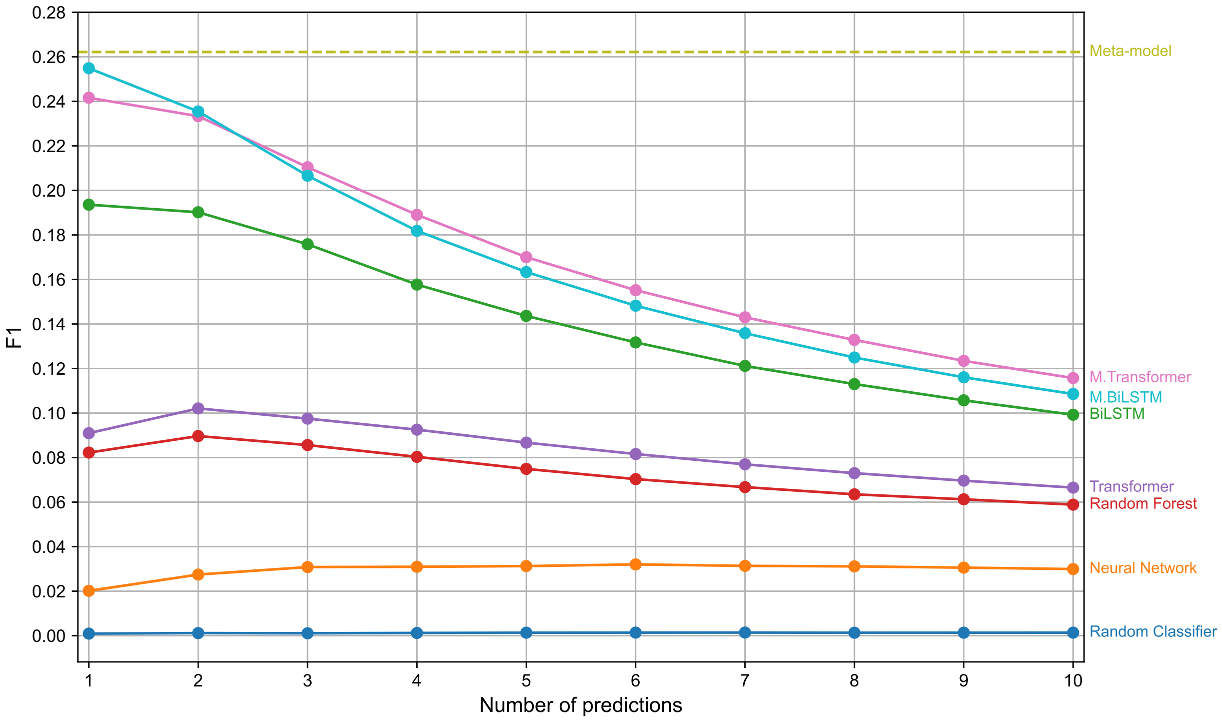

Figure S1: Comparison between the meta-model (dashed line) and the evaluated methods for drug repurposing when varying the number of generated ATC codes per compound in terms of F1 score. The comparison shows that using the meta-model to adapt the number of ATC codes to be generated for each compound yields superior F1 score than fixing in advance the number of ATC codes to be generated.
